# Next-Generation Biomaterials for Vital Pulp Therapy: Exploring Biological Properties and Dentin Regeneration Mechanisms

**DOI:** 10.3390/bioengineering12030248

**Published:** 2025-02-28

**Authors:** Vidhyashree Rajasekar, Mohamed Mahmoud Abdalla, Mengyu Huang, Prasanna Neelakantan, Cynthia Kar Yung Yiu

**Affiliations:** 1 Division of Paediatric Dentistry and Orthodontics, Faculty of Dentistry, The University of Hong Kong, Hong Kong SAR, China; vidhya@connect.hku.hk (V.R.); huangmyu@connect.hku.hk (M.H.); 2 Dental Biomaterials, Faculty of Dental Medicine, Al-Azhar University, Cairo 11651, Egypt; mohamabd@hku.hk; 3 Division of Restorative Dental Sciences, Faculty of Dentistry, The University of Hong Kong, Hong Kong SAR, China; 4 Mike Petryk School of Dentistry, Faculty of Medicine and Dentistry, University of Alberta, Edmonton, AB T6G 2R3, Canada; prasanna.neelakantan@ualberta.ca; 5 Li Ka Shing Institute of Virology, University of Alberta, Edmonton, AB T6G 2R3, Canada; 6 Department of Medical Microbiology and Immunology, University of Alberta, Edmonton, AB T6G 2R3, Canada

**Keywords:** dentin, pulp, biomaterials, regeneration

## Abstract

The advancement of Vital Pulp Therapy (VPT) in dentistry has shown remarkable progress, with a focus on innovative materials and scaffolds to facilitate reparative dentin formation and tissue regeneration. A comprehensive search strategy was performed across PubMed, Scopus, and Web of Science using keywords such as “vital pulp therapy”, “biomaterials”, “dentin regeneration”, and “growth factors”, with filters for English language studies published in the last 10 years. The inclusion criteria focused on in vitro, in vivo, and clinical studies evaluating traditional and next-generation biomaterials for pulp capping and tissue regeneration. Due to the limitations of calcium-based cements in tissue regeneration, next-generation biomaterials like gelatin, chitosan, alginate, platelet-rich fibrins (PRF), demineralized dentin matrix (DDM), self-assembling peptides, and DNA-based nanomaterials were explored for their enhanced biocompatibility, antibacterial properties, and regenerative potential. These biomaterials hold great potential in enhancing VPT outcomes, but further research is required to understand their efficacy and impact on dentin reparative properties. This review explores the mechanisms and properties of biomaterials in dentin tissue regeneration, emphasizing key features that enhance tissue regeneration. These features include biomaterial sources, physicochemical properties, and biological characteristics that support cells and functions. The discussion also covers the biomaterials’ capability to encapsulate growth factors for dentin repair. The development of innovative biomaterials and next-generation scaffold materials presents exciting opportunities for advancing VPT in dentistry, with the potential to improve clinical outcomes and promote tissue regeneration in a safe and effective manner.

## 1. Introduction

Regenerative techniques such as Vital Pulp Therapy (VPT) have gained significant interest in conservative endodontic procedures, with current research focusing on their potential to reconstruct the natural structure, rather than solely acting as a physical barrier [1]. The contemporary goal of regenerative endodontics (RE) is to preserve and restore the health of dental pulp and periapical tissues, ultimately ensuring a functional and healthy dentition in the long term [2]. The holy trinity of regenerative endodontics encompasses the identification of dependable cell sources, signalling molecules to steer tissue regeneration, and more importantly appropriate scaffolds for cellular sustenance and differentiation [3]. Since the dental pulp serves as a treasure chest for the progenitor, dental pulp stem cells (DPSCs), developing an appropriate scaffold with bioactive properties can enable us to mimic the pulp tissue’s innate reparative response and reconstruct the damaged dentin–pulp tissue with native structure and function.

The extracellular matrix (ECM), a vital element of tissue architecture, comprises a fibrous network of proteins and glycosaminoglycans that support and regulate cellular functions. Within these matrices lie embedded protein-like growth factors and signalling molecules that attract progenitor cells and trigger differentiation. Modern approaches involve constructing bioactive scaffolds that imitate the natural extracellular matrices to enhance dentin regeneration. Despite the challenges of replicating the precise structure and function, extracellular matrix (ECM)-inspired scaffolds exhibit exceptional biological characteristics [4]. Key components of a bioactive scaffold imitating native tissue structure include biocompatibility, growth factor encapsulation for differentiation promotion, and favourable physical attributes like biodegradability, viscosity, and tensile strength [5]. Recent research has introduced a variety of materials to develop bioactive scaffolds with excellent biocompatibility. Understanding the mechanisms by which these biomaterials enhance differentiation will enable the development of next-generation biomaterials.

This review aims to explore the mechanisms and biological properties of materials used in dentin tissue regeneration. It covers various biomaterial sources and their influence on physicochemical properties. It also examines the biological characteristics of materials in supporting resident cells and fostering biological functions to stimulate dentin repair and prolong the half-life of bioactive molecules.

## 2. Traditional Dental Pulp Capping Materials: Mechanisms of Action and Biological Properties

Calcium hydroxide (CH) was first introduced in the 1920s as a conventional pulp capping substance, demonstrating an initial success rate ranging between 80 and 90% [6]. However, recent studies show success rates dropping to 58.7–76.3% after 10 years of application [7]. CH stimulates the development of reparative dentin by triggering a superficial layer of coagulation necrosis within the pulp, creating an alkaline environment and increasing calcium ion availability [8]. Despite these benefits, CH has limitations like dissolution within 1–2 years, lack of adhesion to dentin, and tunnel defects, leading to risks of microleakage and reinfection [9]. These drawbacks raise concerns about the long-term efficacy of CH in pulp therapy, despite its antimicrobial properties.

In the early 1990s, mineral trioxide aggregate (MTA), an advanced type of hydrophilic calcium silicate cement (HCSC), was pioneered by Torabinejad and colleagues [10]. Comprising tricalcium silicate, dicalcium silicate, tricalcium aluminate, bismuth oxide, and calcium sulphate dehydrate, the hygroscopic nature of MTA allows it to set effectively in the presence of water, a unique advantage over conventional dental materials [11]. Recent evidence suggests that MTA offers superior clinical outcomes compared to CH, with success rates for direct pulp capping exceeding 90% in some studies [12,13]. MTA’s strong physical properties, antimicrobial effects, and biocompatibility contribute to its favourable clinical performance, maintaining success rates over extended follow-up periods, in contrast to the time-dependent decline observed with CH [14,15]. However, MTA pose some disadvantages, such as discoloration, extended setting time, handling challenges, and higher cost, limiting its widespread application in clinical settings.

Calcium hydroxide- and MTA-based cements pose enhanced differentiation properties on resident DPSCs, modulate the immune response by macrophage polarization (M1 to M2 phase), and have antimicrobial effects against caries pathogens. The primary mechanism through which these cements perform is via the release of Ca^2+^ ions. Although the precise role of released Ca^2+^ ions is not fully understood, studies highlight their importance in dentin hard tissue formation [16]. It was proposed that Ca^2+^ released by biomaterials is a key factor in the mineralization process and actively contributes to reparative dentin formation [17]. The secreted Ca^2+^ skews the expression of the fibronectin gene expression in dental pulp cells, a crucial protein in odontogenic differentiation. Elevated levels of Ca^2+^ also stimulate the differentiation and mineralization of various dental mesenchymal cells, like cementoblasts, through the enhancement of FGF-2 expression [18].

Mechanisms of action for calcium-based cements involve the conversion of latent bioactive molecules into their active forms. Growth factors within the extracellular matrix often start in a latent state, needing activation by signalling molecules. Research indicates that the release of Ca^2+^ generates a highly alkaline environment that triggers the conversion of these bioactive substances into their active states. This phenomenon was demonstrated specifically in relation to TGF-β [19]. Ca^2+^ could potentially create an alkaline environment, aiding in the conversion of TGF-β to its active form. While direct evidence in dental tissue is lacking, a recent in vitro study examined the combined effect of TGF-β and CH on TGF-β production by osteoblasts. The study revealed that although the difference was not statistically significant, the combination consistently produced higher levels of TGF-β compared to TGF-β alone. This suggests that Ca^2+^ synergistically enhances the bioactivity of TGF-β on osteoblasts [20].

While calcium-based cements are essential for releasing active growth factors and driving differentiation, they can also induce cytotoxic effects. Reports indicate that the pH in the microenvironment influenced by released Ca^2+^ can rise as high as 12.5, exceeding the optimal survival range for odontoblasts [21]. Alkaline phosphatase, abundant at mineralization sites, aids in hydrolysing inhibitory molecules like pyrophosphate ions, while also providing essential inorganic phosphate for mineral crystal formation. The optimal pH for alkaline phosphatase activity in laboratory settings is around 10 [22]. Notably, the alkaline nature can inhibit the pyrophosphate ions, key inhibitors of dentin tissue formation. The application of CH-containing pulp capping agents to pulp can create the required alkaline environment for hard tissue formation. The increased alkalinity establishes an unfavourable environment for bacterial growth, providing calcium-based cements with significant pathogen-clearing capabilities. However, this antibacterial effect is mostly limited to the surface layers of the injury, rendering the antibacterial properties effective primarily against minimal bacterial invasion [23,24]

## 3. Next-Generation Scaffold Materials

Biomaterials can be classified according to their source as either natural or synthetic. Natural biomaterials, such as protein-like collagen, exhibit inherent biocompatibility and biodegradability. Nevertheless, their degradation rate and mechanical characteristics often require enhancement through modifications. In contrast, synthetic scaffolds are fabricated and can be categorized into polymeric, bio-ceramic, or metallic materials.

### 3.1. Natural Biomaterials and Their Application in VPT

Natural biomaterials display significant advantages over the synthetic and bio-ceramic-based biomaterials. These natural polymers are often derived from the ECM proteins. Fibronectin, laminins, tenascins, collagen, and vitronectins play roles in various cellular functions like growth, differentiation, and migration [25]. The proteins have a crucial domain for integrin attachment to cell membranes, initiating essential molecular signalling pathways. Apart from biochemical signals, the mechanical properties (stiffness, elasticity) of the extracellular matrix (ECM) also influence cell behaviour. Cell response to the ECM is influenced by factors including ECM type, concentration, mechanical and physical properties, ECM adsorption method, and the presence of other proteins. ECM proteins, typically synthesized and released by fibroblasts and other cell varieties like epithelial cells, have crucial functions in cellular proliferation and maturation within living organisms [26].

#### 3.1.1. Collagen and Gelatin

Collagen, the primary structural protein in the extracellular matrix of connective tissues, closely mimics the physical properties of real pulp tissue [27]. Comprising amino acid sequences like glycine-X-Y, where X and Y are often hydroxyproline or proline, collagen forms a triple helix structure [28]. Collagen is sourced from different animal or human tissues such as cartilage, bone, ligament, tendon, or skin, with low immunogenicity due to its natural origin. It exhibits permeability, a porous nature, biocompatibility, and biodegradability, which influence cellular behaviour such as morphology, adhesion, differentiation and migration. These attributes position collagen as a potential biomaterial and scaffold for tissue engineering. Nevertheless, challenges like inadequate mechanical strength and structural stability upon hydration are present, affecting its usability [29]. Improvements can be made by crosslinking collagen scaffolds and combining collagen with other materials, such as inorganic substances or natural/synthetic polymers, to effectively enhance mechanical properties [30]. Collagen and its derivative gelatin are proven to have excellent biological and mechanical properties to be developed as a pulp capping material. Numerous research investigations have utilized off-the-shelf collagen and gelatin sponges as easily accessible biomaterials to assess how effective bioactive substances are in improving dentin restoration [31,32].

Gelatin polymer, a well established biodegradable and biocompatible material, contains around 85–92% proteins, water, and mineral salts. Gelatin is produced by irreversibly breaking down collagen’s triple helical structure through methods like heat and enzymatic denaturation, leading to the formation of disordered coiled structures [33]. Although less organized in structure, gelatin closely resembles collagen at a molecular level, allowing it to effectively replicate collagen in diverse biomaterial applications for cellular growth. Gelatin sourced from different origins has shown its general biocompatibility with human cells, with no significant toxicity or antigenicity observed [34]. However, the use of specific crosslinking agents in gelatin solutions can introduce potential toxicity risks. A key characteristic of gelatin-based biomaterials is its biodegradability, triggered by gelatinases (MMP2 and MMP9). These enzymes are often present in the inflamed microenvironment, aiding in the breakdown of gelatin polymers and the release of encapsulated bioactive components to promote tissue repair [35]. Gelatin and collagen are not directly approved by the FDA as standalone substances, but are widely recognized as safe and compliant when used in FDA-regulated products such as pharmaceuticals, medical devices, and food additives [36]. 

Gelatin methacryloyl (GelMA), a material derived from modified gelatin with methacrylate groups, is increasingly recognized for tissue engineering applications due to its affordability, tunability, compatibility with cells, and ability to be crosslinked using light. GelMA hydrogel can be solidified using light in the presence of a photo-initiator that generates free radicals. Acrylate groups within the gelatin structure participate in chain reactions initiated by radicals [37]. Typically, GelMA hydrogels contain less than 5% methacryloyl groups (MA), ensuring that crucial amino acid motifs like RGD and MMP within gelatin remain largely unaffected by the presence of MA. The RGD sequence, essential for cell adhesion, remains unaltered as it does not interact with MA, thereby preserving gelatin’s effectiveness as a material for promoting cell attachment [38]. GelMA hydrogel shows promise as a material for constructing scaffolds for a range of tissues, including those in dental, orofacial, and skeletal domains, in forthcoming clinical scenarios.

GelMA has been combined with additional functional materials to induce reparative, antibacterial, and immunomodulatory effects, as detailed in Table 1. Many studies have incorporated gelatin or GelMA with tricalcium silicate/other calcium silicate cements to transform cement-based materials into an injectable form for simplified clinical application. Combining gelatin/GelMA with antimicrobial, angiogenic, and tissue regenerative compounds has shown the ability to mitigate the conditions present in injured dental pulp. Although studies demonstrate the efficacy of collagen/gelatin-based scaffolds in pulp–dentin regeneration, their application is mainly limited to in vivo settings. Achieving authentic pulp tissue regeneration is complex. Despite the promise of GelMA hydrogels for their injectability and biocompatibility, they struggle to replicate the intricate pulp tissue microenvironment crucial for successful regeneration [39]. Variations in gelatinase profiles among highly proliferative/multipotent DPSCs lead to differing degradation patterns of biomaterials [40]. While collagen- and gelatin-based materials hold promise for pulp regeneration, challenges in mimicking natural tissue conditions, stem cell population diversity, and the demand for stronger clinical evidence impede their widespread use in VPT. Addressing these challenges through future research will aid in the development of promising biomaterials for VPT. Table 1 enumerates studies investigating the use of collagen and gelatin as materials for pulp capping procedures.

#### 3.1.2. Chitosan

Chitosan macromolecules have gained significant attention over the past three decades in both research and industrial applications [49]. Its unique chemistry featuring three reactive functional groups—amino/acetamido groups and primary and secondary hydroxyl groups—allows for a wide range of potential chemical modifications and substitutions of functional groups (e.g., OH, NH_2_ with -COCH_3_, -CH_3_, -CH_2_COOH, SO_3_H, -PO(OH)_2_, etc.) [50]. Chitosan and its derivatives were identified as non-toxic, biocompatible, osteogenic, antibacterial, biodegradable, bioresorbable, antioxidant, immunoenhancing, and anti-cancer agents [51]. Moreover, they have enhanced pulp cell properties such as adhesion, differentiation, and proliferation, crucial processes in tissue regeneration. Consequently, chitosan stands out as one of the most extensively studied polymers in tissue engineering for the replacement or restoration of damaged organs or tissues [52]. Chitosan’s FDA approval enables its versatile application, especially in drug delivery and biomedical research. This endorsement highlights its promising role as a secure and efficient biomaterial within these fields [53].

An intriguing mechanism of chitosan in mineralization involves its role in dentin remineralization, which is categorized into two types: intra- and extra-fibrillar remineralization, also known as guided tissue remineralization (GTR). During GTR, the process enhances the mechanical properties of dentin by relying on a non-collagenous protein known as dentin matrix protein 1 (DMP1) [52]. DMP-1 acts as a mediator between Ca^2+^ ions and hydroxyapatite crystals [54]. Analogous proteins, such as carboxymethyl chitosan (CMC), can replace DMP1 and fulfil the same function. Santoso et al. utilized CMC/ACP for dentin remineralization, demonstrating a significant enhancement in the remineralization process and strengthening of dentin [55]. In a study by Annisa RN et al., [52] CMC/ACP was employed to mimic the role of DMP1. The efficacy of CMC/ACP in achieving both intra- and extra-fibrillar remineralization on demineralized dentin was investigated. The results showed a notable increase in calcium and phosphate ion levels within CMC/APC crystals from day 7 to day 14. Transmission electron microscopy analysis revealed that the CMC/ACP group exhibited enhanced intra- and extra-fibrillar remineralization compared to other remineralization agents [56].

A randomized controlled trial involving participants aged 15–45 with necrotic single-rooted teeth compared native chitosan-based, enzymatically modified chitosan (EMCS)-based, and blood clot (BC) scaffolds. While no significant differences were observed in healing at one, three, and twelve months, the EMCS+BC group showed superior healing at the six-month mark. The study indicates that the EMCS+BC scaffold could enhance pulp regeneration outcomes after six months, underscoring the critical role of scaffold selection in regenerative endodontics [57]. Another study presented a protocol for evaluating the dentin–pulp bio-stimulation chitosan membrane (BBio) in VPT for primary teeth. This randomized clinical trial aims to evaluate the clinical and radiographic outcomes of this chitosan-based material compared to MTA over a follow-up period of 12 to 24 months. The study emphasized the potential of chitosan in preserving pulp vitality and stimulating tissue regeneration [58].

While these studies underscore the potential of chitosan-based materials in pulp capping, further clinical trials are imperative to confirm their efficacy and long-term outcomes compared to conventional materials. Previous research suggests that chitosan may offer beneficial properties for pulp capping, but comprehensive studies are needed to validate these findings and optimize its clinical application. The studies on the application of chitosan as a pulp capping agent over the past 5 years are listed in Table 2.

#### 3.1.3. Alginate

Despite alginate’s unique properties making it attractive for various biomedical applications and tissue engineering, it also comes with significant drawbacks. The dissolution of alginate gels is primarily caused by interactions between monovalent cations and alginate blocks. While gels formed with alginate dissolve under physiological conditions in mammals, the alginate molecules cannot be completely eliminated from the body due to their average molecular mass, exceeding the kidney’s renal clearance threshold [75,76]. Additionally, alginate’s lack of cell adhesion capabilities leads to poor cell attachment and reduced interactions between cells and the material in both 2D and 3D environments [77]. Most of the studies used alginate in conjunction with other polymers or supplemented with bioactive molecules to enhance adhesion and other biological properties [66,67,68]. One key advantage of chitosan over alginate is its notable antibacterial properties, particularly effective against various important bacterial and fungal species found in the oral cavity.

#### 3.1.4. Platelet-Rich Fibrins

Platelet-rich fibrin (PRF) is acquired through centrifugation without the use of anticoagulants, making it entirely autologous. This fibrin matrix comprises platelets, leukocytes, and an array of cytokines and growth factors, such as IL-1β, IL-4, IL-6, TGF-β1, PDGF, and VEGF [78]. As the coagulation cascade produces fibrin and platelets release cytokines, PRF becomes a highly biocompatible matrix, particularly beneficial in injured areas where the fibrin network also serves as a reservoir for tissue growth factors. These elements play a direct role in stimulating the growth and specialization of osteoblasts, endothelial cells, chondrocytes, and various types of fibroblasts. However, uncertainties persist regarding the actual clinical effectiveness of PRF [79].

The concentrated platelet-rich growth factor suspension in PRF stimulates tissue regeneration and wound healing. TGF-β boosts dentin formation by enhancing odontoblastic activity. Additionally, leukocytes, cytokines, and lymphocytes combat infections and inflammation. VEGF supports angiogenesis crucial for revascularization. Various cytokines, including FGF, VEGF, angiopoietin, and PDGF, are trapped in the fibrin matrix, gradually released to aid in angiogenesis. Fibrin enhances αvβ3 integrin expression, facilitating endothelial cell binding to fibrin and other proteins. It aids in cell adhesion and the movement of immune cells. Fibrin also increases CD11c/CD18 receptor expression on endothelial cells, and influences macrophage presence in wounds. The fibrin scaffold supports undifferentiated mesenchymal cells, promoting their differentiation, which is essential for tissue regeneration [80].

Platelet-rich plasma is a prominent pulp capping material because of its excellent tissue compatibility and antibacterial characteristics. Its capacity to enhance mineralization, cell proliferation, and attract DPSCs in pulp makes it a promising option for direct pulp capping [80]. Moreover, PRP is an affordable, efficient, safe, and sterile method, as it can be promptly derived from the patient’s blood [81].

A recent study has found that PRP promoted the mineralized dentin bridge formation, after direct pulp capping (DPC), akin to MTA, which is considered the benchmark [82]. Platelet-rich plasma has the potential to elicit superior cellular dentin-forming reactions and facilitate the restoration of dentin with a consistent structure compared to MTA in canine teeth. Additionally, in the PRP-treated group, there was a marked increase in the expression of dentinogenesis-related genes such as DSPP, MEPE, and NES mRNA when compared to the MTA-treated group [83].

Platelet-rich fibrin (PRF) demonstrates significant clinical potential in dentistry, particularly as a pulp capping material, due to its ability to promote tissue regeneration and healing [82]. PRF is biocompatible, cost-effective, and avoids ethical concerns associated with xenogenic materials like fetal bovine serum, making it suitable for stem cell cultures and regenerative applications [84]. Advantages include ease of preparation, sustained growth factor release, and reduced immune rejection risks [84]. However, limitations include underutilization in pediatric dentistry due to its blood-derived nature, lack of standardized protocols for optimal concentration in cell culture, and limited long-term data. While clinical assessments show comparable efficacy to MTA and PRP in pulp capping, PRF’s regenerative properties position it as a promising alternative for preserving pulp vitality, particularly when combined with bioactive materials [85]. Further research is needed to address its constraints and validate its broader clinical adoption. The studies on the application of PRF as a pulp capping agent over the past 5 years are listed in Table 3.

#### 3.1.5. Demineralized Dentin Matrix

The concept of replacing the lost tissue involves mimicking the original structure and function of the native tissue to maintain homeostasis. This idea holds significant value in the context of dentin repair and regeneration. It is crucial to achieve the seamless integration of newly formed dentin tubules with the existing dentin for the revitalization of the dentin–pulp complex. The distinctive tubular structure of dentin, which contains odontoblasts and nerve endings, not only provides mechanical strength to the tooth but also plays a crucial role in immune responses and sensitivity functions. To achieve optimal dentin regeneration, the most effective approach is to utilize a scaffold that closely mimics dentin tissue [90].

The dentin matrix serves as a natural framework for both dentin and bone tissue engineering. Various dentin-derived materials were developed from human or animal dentin using diverse processing techniques. Typically, demineralized dentin matrix (DDM) involves the removal of cementum and enamel, followed by dentin pulverization and demineralization using EDTA or hydrochloric acid for different durations. Demineralized dentin is created by fully or partially demineralizing fresh dentin through specific protocols, resulting in DDM powders ranging from 300 to 800 μm after grinding [91,92]. Throughout this processing, the fundamental structures, such as dentin tubules, peritubular dentin, and intratubular dentin, is generally retained [90]. Previous research on the clinical application of DDM, with a 24-month follow-up study, demonstrated that dentin formation was significantly greater compared to MTA and Biodentine. Since the nature of DDM is in powder form, it is always used with other polymers such as gelatin, alginate, and chitosan [93].

A study highlighted that although DDM exhibited a success rate of 92.86% in apexification procedures, similar to calcium hydroxide, the difference lacked statistical significance, underscoring the need for further investigation to ascertain its clinical efficacy [94]. Additionally, significant hurdles persist in VPT, notably uncontrolled infections and excessive inflammation, posing substantial challenges [47]. The full clinical potential of demineralized dentin matrix (DDM) in VPT remains largely unexplored, despite its recognized advantages. The use of DDM in VPT is listed in Table 4.

### 3.2. Synthetic Polymers

Synthetic materials offer advantages by enabling greater control over scaffold microarchitecture and mechanical properties. They are also cost-effective compared to natural biomaterials [97]. However, synthetic materials are more prone to triggering inflammatory and immunogenic reactions or generating toxic by-products [98]. Hybrid scaffolds combine various biomaterials to improve the physical and mechanical properties of natural biomaterials while mitigating the biocompatibility issues associated with synthetics [99].

#### 3.2.1. Self-Assembling Peptides

Since the late 1980s, there has been a growing interest in short peptides derived from natural proteins that possess self-assembling properties. Over time, a multitude of engineered peptides have been developed to mirror the self-assembling characteristics of those found in protein molecules [100]. The assembly process, typically triggered by external factors, relies on secondary structures like β-sheets and α-helices [101]. Factors such as ionic strength, pH, and temperature are commonly employed to initiate the formation of complex nanostructures [102]. Self-assembling peptide (SAP) hydrogels respond to these stimuli, showing promise for biomedical uses due to their compatibility, degradability, facile functionalization, and similarity to the extracellular matrix. Moreover, the primary structure of amino acids provides attachment sites for polymeric substrates [100]. Beyond self-assembly, these peptides can easily exhibit additional properties like self-healing, shear-thinning, and shape memory [103]. Self-assembling peptides have the capability to imitate the natural biomechanics and structure of tissues, as well as the ECM. In addition to their inherent framework, SAPs can be adjusted to incorporate biologically active components. The advantages of SAP have garnered significant attention in the field of biomedical research, including dentistry [104].

RADA16, an SAP that was commercialized and is undergoing clinical trials, is known for its significant tissue regenerative properties [105,106]. RADA16 sequences, influenced by the common presence of the adhesion-promoting RGD motif in proteins like fibronectin, were designed to enhance cell adhesion and survival in laboratory cultures. RADA16, marketed as PuraMatrix^TM^ by 3-D Matrix Ltd. in Tokyo, Japan, is a 1% peptide solution employed as a substitute for the extracellular matrix (ECM) in laboratory studies related to cell adhesion, chemotaxis, proliferation, and growth. During clinical procedures or in the operating room, the SAP items PuraStat^®^ and PuraBond^®^ (3-D Matrix Europe SAS, Caluire-et-Cuire, France) are administered through prefilled syringes as thick aqueous solutions containing synthetic 2.5% RADA16, which are applied to cover the wound surface [97,106]. RADA16 has been extensively studied for its hard tissue reparative effects and pulp regenerative models, but its application for dentin reparative effects and pulp capping efficiency has not been explored yet. Studies have shown that RADA16 houses DPSCs with high biocompatibility, without causing side effects such as immunogenicity [107]. While the impact of RADA16 on dentin repair effects remains relatively unexplored, this substance shows great potential for use in Vital Pulp Therapy.

Another self-assembling peptide, in addition to RADA16, has gained regulatory approval for clinical applications. This peptide, consisting of 11 amino acids (QQRFEWEFEQQ) and named P11-4 (sold as CurodontTM Repair), facilitates remineralization and reversal of carious lesions, according to various studies [108,109]. The self-assembling peptide P11-4 is a biomimetic protein designed to replicate the native process of enamel remineralization. By binding to demineralized enamel surfaces, P11-4 creates a three-dimensional network that supported mineral deposition, aiding in enamel remineralization. Additionally, P11-4 stimulates the production of reparative proteins to help restore damaged enamel [5]. This non-invasive treatment has the potential to transform dental caries management [110]. P11-4 also proven to prevent the collagen proteolysis in dentin [111]. Preclinical and clinical studies have demonstrated the efficacy of P11-4, with various trials comparing its effects to other remineralizing agents in treating early stages of carious enamel or white spot lesions [112].

In vitro experiments, with assembled P11-4 in “mineralizing” solutions, revealed the formation of needle-like electron-dense deposition with poor crystalline hydroxyapatite formation. This suggests that the enhanced mineral accumulation in the lesions could be attributed to mineral precipitation within the assembled scaffold on site. However, further confirmation is needed as no analysis of the mineral content within the lesions has been conducted [112]. The peptide was shown to enhance the osteogenic differentiation of stem cells from apical papillae (SCAP). Nevertheless, this peptide has primarily been investigated for its remineralization characteristics. Further exploration of the biological impacts of these peptides on pulp resident cells will allow us to refine them into a potential material for pulp capping.

Self-assembling peptides (SAPs) in VPT have drawbacks, as highlighted in various research. Concerns include varied effectiveness in promoting remineralization and tissue regeneration. For example, P11-4 may not significantly improve enamel microhardness compared to other treatments, limiting its efficacy in dental caries management [113]. The clinical potential of SAPs needs more exploration across diverse dental diseases for reliable adoption in VPT. Incorporating SAPs into tissue engineering scaffolds shows promise, but outcomes are influenced by scaffold architecture [111,112]. Various 3D scaffold geometries affect cell behaviour, impacting SAP success. While SAPs can aid cell recruitment and angiogenesis, challenges in dentin–pulp regeneration like disinfection and tissue regrowth may limit overall regenerative results. In conclusion, while SAPs offer innovative approaches in VPT, their efficacy limitations, varied clinical outcomes, and reliance on scaffold design require further investigation [114].

#### 3.2.2. DNA-Based Nanomaterials

DNA possesses ideal chemical properties for self-assembly at the molecular level. The specificity of Watson–Crick base pairing permits numerous DNA strands to autonomously form intricate 3D nano- or microstructures [115]. Within hybrid DNA hydrogels, short DNA segments are attached to polymer frameworks to act as both connectors and modifiable components. The formation of secondary structures in self-assembled DNA relies on multiple interactions like hydrogen bonding, hydrophobic interactions, and π-π stacking, resulting in DNA hydrogels with superior strength compared to those based on a single supramolecular interaction [116]. Additionally, the wide array of DNA sequence designs enables these hydrogels to display diverse responsiveness and functionalities. For instance, a DNA–carbon nanotube (CNT) composite hydrogel, achieved by linking a cytosine-rich DNA sequence to CNTs through π-π stacking, showed sensitivity to pH changes and adjustable mechanical properties [117].

Similarly to SAP, the DNA-based nanostructure also offers fine tunability in its mechanical and chemical properties. The facile synthesis and functionalization properties of this material make it an ideal candidate for tissue engineering applications. The highly negative charge density of DNA structures makes them strongly attracted to cations relevant for biomimetic mineralization processes. Single-stranded DNA (ssDNA), double-stranded DNA (dsDNA), and self-assembled DNA frameworks are integral to novel mineralization techniques that offer a wide array of morphologies [118]. DNA assemblies are particularly well suited for directing biomineralization as they can maintain structural order across scales ranging from nanometers to millimetres, crucial for regenerating mineralized tissues. In biomimetic mineralization, ions such as Ca^2+^, CO_3_^2−^, PO_4_^3−^, and Si(OEt)_4_ (tetraethoxysilane, TEOS—for generating SiO_2_) play essential roles in producing the inorganic materials present in mineralized tissues. The crystallization process is regulated by the ionic interactions of SiO_2_ precursors or Ca^2+^ with the anionic DNA sugar-phosphate backbone, resulting in structures that resemble natural biominerals such as tooth enamel and bone [119]. By employing synthetic techniques to create biomimetic minerals for hard tissue (bone and teeth), valuable insights into tissue regeneration can be gained [120]. These methods also enable the development of biomolecule–inorganic hybrid materials with advantageous properties. Very limited research has been reported on the biomineralization properties of DNA-based nanomaterials and their specific influence on dentin regeneration remains unexplored. Extensive in vitro studies are required to thoroughly investigate DNA-based materials, as their progression toward clinical application is still in its early stages.

#### 3.2.3. Other Synthetic Polymers

Synthetic polymers represent the largest group of biodegradable polymers. When manufactured in controlled environments, these synthetic polymeric scaffolds exhibit consistent and predictable properties such as viscosity, porosity, and biodegradation rates. The controlled production of these scaffolds allows for tunability in terms of their physical and chemical attributes, enabling the incorporation of growth factors and other bioactive molecules. Synthetic polymers can be mass-produced, potentially offering cost advantages over natural scaffolds and longer shelf lives. However, their hydrophobic nature limits their bioactivity. Popular synthetic polymers in tissue engineering include polylactic acid (PLA), polyglycolic acid (PGA), polycaprolactone, and polylactide-co-glycolide (PLGA). The FDA approval of these synthetic polymers for medical device applications paves the way for the development of a biomaterial that can be readily integrated into clinical practice [25,121].

While the synthetic polymers satisfy the criteria for engineering the scaffold with critical physical properties that mimic ECM, they lack bioactivity by themselves. As discussed earlier, the natural compound can promote adhesion through the RGD domains and enzyme-responsive behaviours. Although these synthetic polymers may lack biological properties, they provide an excellent option for loading the scaffold with growth and tissue reparative proteins. The combination of synthetics scaffolds with calcium phosphate ceramics were proven to overcome issues such as brittleness, dissolution, and modelling efficiency. By combining synthetic polymers with natural compounds that provide bioactivity, such as RGD domains and enzyme-responsive behaviours, a synergistic effect can be achieved, which enhances the regenerative potential of the scaffold. Combining synthetic polymers with calcium phosphate ceramics improves the mechanical strength and bioactivity of the scaffold, offering a promising approach for dentin regeneration in VPT. By integrating synthetic and natural components strategically, customized synthetic polymer scaffolds can play a crucial role in promoting dentin repair and tissue regeneration, demonstrating their effectiveness in advancing pulp capping agents [25,121].

## 4. Conclusions and Future Perspectives

In conclusion, the field of regenerative endodontics is rapidly progressing, emphasizing the development of innovative strategies for dentin tissue regeneration in VPT. While conventional pulp capping materials like MTA, CH, and Biodentine have found clinical success, limitations such as discoloration, prolonged setting time, and inadequate tissue regeneration have led research into novel materials with improved physical and biological properties. Advanced and biocompatible substances such as collagen, gelatin, chitosan, PRF, and DDM are under investigation for their efficacy as pulp capping agents, either alone or in combination. Although these materials demonstrate dentin regenerative effects, current research primarily focuses on their applications in laboratory settings and animal models. Materials such as collagen/gelatin-based scaffolds, chitosan-based materials, PRF, and DDM showcase promising potential for enhancing dentin–pulp regeneration. While these materials offer unique advantages, challenges persist in replicating the intricate pulp tissue microenvironment and establishing robust clinical evidence for widespread adoption. Further research, particularly through randomized controlled trials and long-term studies, is essential to validate the efficacy and safety of these biomaterials in diverse clinical scenarios. Additionally, exploring the applications of SAPs and DNA-based nanostructures in VPT presents exciting opportunities for fine-tuning the mechanical and chemical properties, directing biomineralization processes, and advancing tissue regeneration strategies. Comprehensive investigations into these innovative biomaterials are crucial for their application in regenerative endodontics and to advance them closer to clinical use.

## Figures and Tables

**Table 1:** Studies on collagen and gelatin as pulp capping materials.

S. No.	Materials	Effect on DPSCs	Dentin Repair—In Vivo	Antibacterial Effects	References
1.	GelMA with tricalcium phosphate (TCP) nanocomposite	Promoted odontogenic differentiation of DPSCs	Enhanced dentin repair	-	[41]
2.	SrCuSi_4_ O_10_/GelMA composite hydrogel	Promoted odontogenic differentiation	Enhanced dentin repair	Antibacterial and anti-biofilm effect against *S. mutans* and *L.casei*	[42]
3.	Dentin extracellular matrix-loaded bioactive glass/GelMA	Promoted odontogenic differentiation	-	-	[43]
4.	Platelet lysate functionalized gelatin methacrylate microspheres	Improved proliferation and odontogenic differentiation of DPSCs	Subcutaneous implantation into SCID mice-enhanced angiogenesis, pulp-like tissue formation	-	[44]
5.	Methacrylated gelatin/thiolated pectin hydrogels with melatonin/tideglusib-loaded core/shell PMMA/silk fibroin electrospun fibres	Promoted odontogenic differentiation	-	-	[45]
6.	Injectable decellularized dental pulp matrix/gelatin hydrogel microspheres	Increased odontogenic differentiation	Tooth slices: Nude mice- promoted pulp–dentin formation with rich blood vessels and odontoblasts	-	[46]
7.	Antimicrobial peptide- and dentin matrix-functionalized gelatin hydrogel	Reduced inflammatory responses and prompted odontogenic differentiation of inflamed hDPSCs	Mice-induced M2 macrophage phenotype Beagle dogs: Promoted pulp–dentin formation	Antibacterial effect against *L. casei* and *A. naeslundii*	[47]
8.	Collagen sponges with regenerative peptide and molecules—Tideglusib, S100A8, and S100A9	Enhanced odontogenic differentiation	Enhanced dentin reparative effect	-	[31,32]
9.	Beta-tricalcium phosphate (β-TCP), hydroxyapatite (HA), and collagen scaffold with mineral trioxide aggregate (MTA)	-	Enhanced reparative dentin formation	-	
10.	Phosphophoryn/ collagen composite	-	Enhanced reparative dentin formation	-	[48]

**Table 2:** Studies on chitosan as pulp capping material.

S. No.	Materials	Effect on DPSCs	Dentin Repair—In Vivo	Antibacterial Effects	References
1.	Tideglusib-hyaluronic acid hydrogels with Rg1-loaded chitosan microspheres	Promoted odontogenic differentiation	-	-	[59]
2.	A double-layer bovine BPB/chitosan: calcium hydroxide	-	Enhanced dentin repair in the presence of calcium	-	[60]
3.	Porous chitosan scaffolds (SCH) with calcium silicate (CaSi)	Enhanced odontogenic differentiation of DPSCs	-	-	[61]
4.	Porous chitosan/calcium/aluminate scaffold (CH-AlCa)with 1α,25-dihydroxyvitamin D3 (1α,25VD)	Enhanced odontogenic differentiation of DPSCs	-	-	[62]
5.	Endometrial stem cells were put on a three-dimensional (3D) chitosan scaffold containing TiO_2_ NPs	-	Enhanced dentin regeneration	-	[63]
6.	Silver-doped bioactive glass/chitosan hydrogel	Promoted odontogenic differentiation of DPSCs	Enhanced dentin repair	-	[64]
7.	VEGF-loaded chitosan hydrogel	Promoted odontogenic differentiation	-	*-*	[65]
8.	Carboxymethyl chitosan (CMC)/amorphous calcium phosphate (CMC/ACP)	CMC facilitated formation of ACP nano-precursors by chelating capacity Tooth model of deep caries Excellent remineralization capacity	-	-	[66,67,68]
9.	RGD-alginate/laponite (RGD-Alg/Lap) hydrogel micropsheres	Promoted odontogenic differentiation	Promoted neovascularization	-	[69]
10.	Collagen alginate with hydroxyapatite	Promoted odontogenic differentiation of DPSCs	-	-	[70]
11.	Native and enzymatically modified chitosan with blood clot	-	Enzymatically modified chitosan enhanced dentin repair	-	[71]
12.	Dexamethasone-loaded chitosan sponge	Increased odontogenic differentiation of hDPSCs	Renewed dentin bridge, with minimal inflammatory response	-	[72]
13.	Porous chitosan scaffold mixed with calcium cements	Increased odontogenic differentiation of hDPSCs	-	-	[73]
14.	CMC mediated intrafibrillar mineralization	Remineralized artificial caries affected dentin	-	-	[74]

**Table 3:** Studies on PRF and PRP as pulp capping materials.

S. No.	Materials	Dentin Repair—In Vivo and In Vivo	Antibacterial Effects	Clinical Study	References
1.	PRF	-	-	12 month success rate: 82.6% for PRF and 61.9% for MTA	[86]
2.	PRF	Showed increased pulp viability and MTA	-	-	[87]
3.	PRP and PRF			Randomized controlled trial: Significantly higher volume of dentin formed than MTA	[82]
4.	PRF	Increased odontogenic differentiation of DPSCs	-	-	[88]
5.	PRF	-	-	Mandibular molar teeth with irreversible pulpitis: MTA, PRF, and Ca(OH)_2_ showed similar increase in dentin deposition	[89]

**Table 4:** Studies on natural tissue-based polymers as pulp capping materials.

S. No.	Materials	Effect on DPSCs	Dentin Repair—In Vivo	Antibacterial Effects	References
1.	Gelatin methacryloyl microgels (7% *w*/*v*) mixed with dentin matrix molecules	-	Pulp tissue formation, newly formed tubular and atubular dentin, and blood vessel formation	-	[90]
2.	Treated dentin matrix hydrogel (TDMH)	-	Induced dentin bridge formation	-	[91,92]
3.	Photocrosslinkable gelatin-treated dentin matrix hydrogel	-	Increased dentin repair	*-*	[93]
4.	Digested dentin matrix extract (DDME)	Induced proliferation, differentiation of DPSCs	Regenerated dentin in an in situ animal model	-	[95]
5.	Sodium alginate with DDM	DPSCs showed enhanced *COL-1* gene expression	-	More enhanced antimicrobial effect than MTA	[96]
